# Supplementary material for: Vitamin D Receptor Gene Expression in Adipose Tissue of Obese Individuals is Regulated by miRNA and Correlates with the Pro-Inflammatory Cytokine Level
Source: Int J Mol Sci. 2019 Oct 24;20(21):5272. doi: 10.3390/ijms20215272 (PMC6862513; doi:10.3390/ijms20215272)
Supplement: Supplementary file 1 [file ijms-20-05272-s001.pdf]

Supplementary Figure 1

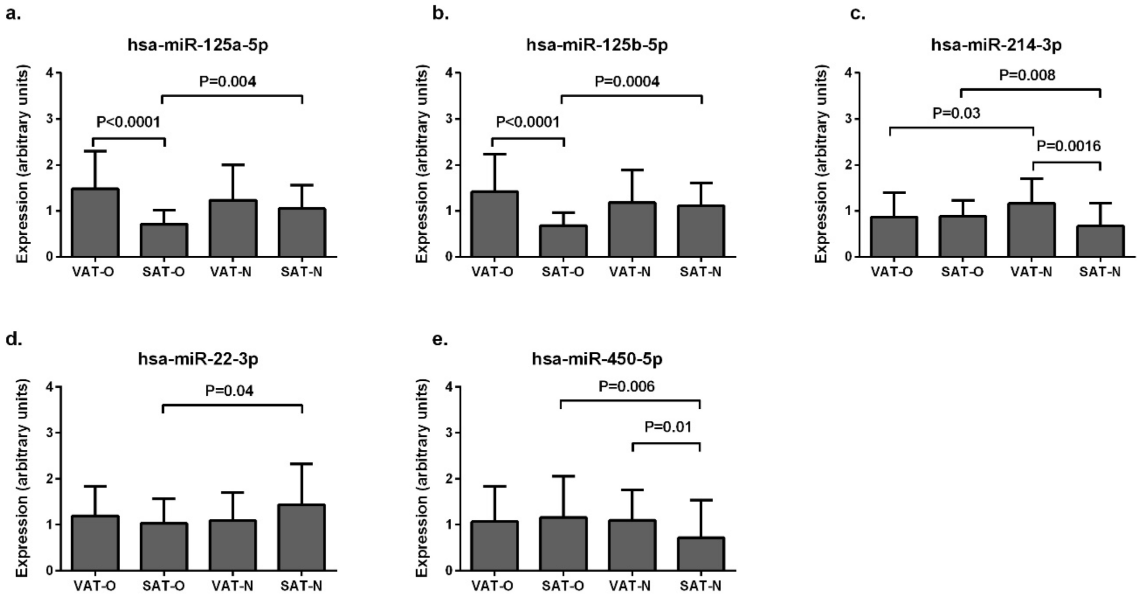

Supplementary Figure 1. Expression of hsa-miR-125a-5p (a), hsa-miR-125b-5p (b), hsa-miR-214-3p (c) hsa-miR-22-3p (d) and hsa-miR-450b-5p (e) in visceral (VAT) and subcutaneous (SAT) adipose tissue samples from the obese (O) and normal-weight (N) individuals. Results, normalized against the expression of hsa-miR103a-3p, are presented in arbitrary units (AU) as mean miRNA levels.

Supplementary Figure 2

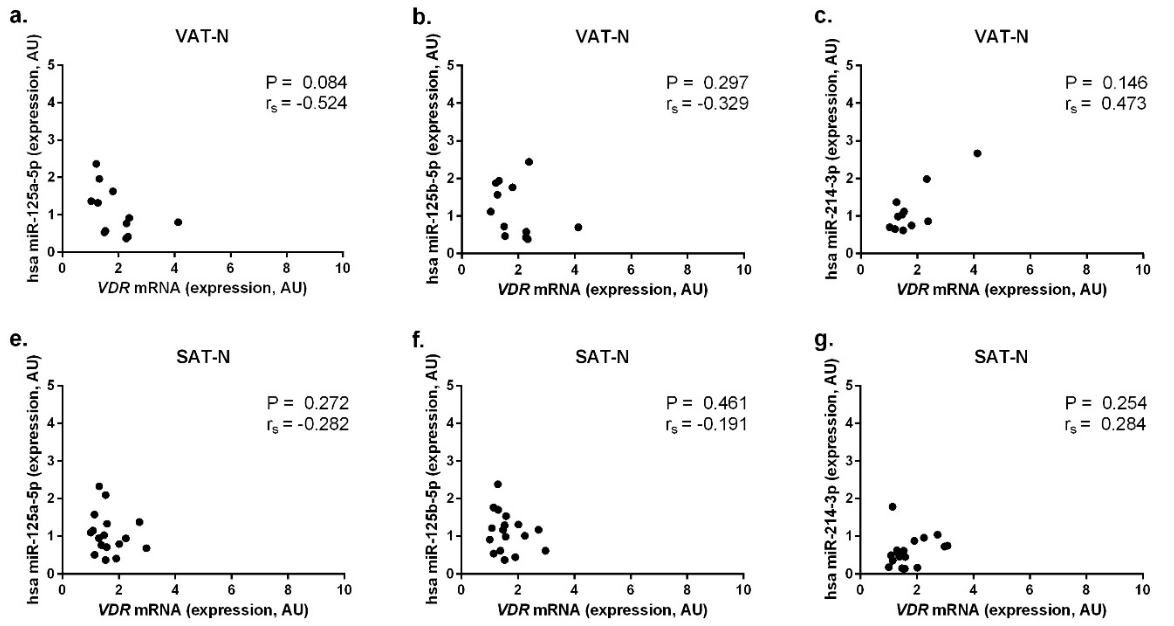

Supplementary Figure 2. Correlation between mRNA levels of *VDR* and of hsa-miR-125a-5p, hsa-miR-125b-5p and hsa-miR-214-3p in visceral (VAT a, b, c, respectively) and subcutaneous (SAT d, e, f, respectively) adipose tissues of normal-weight (N) individuals.

Supplementary Figure 3

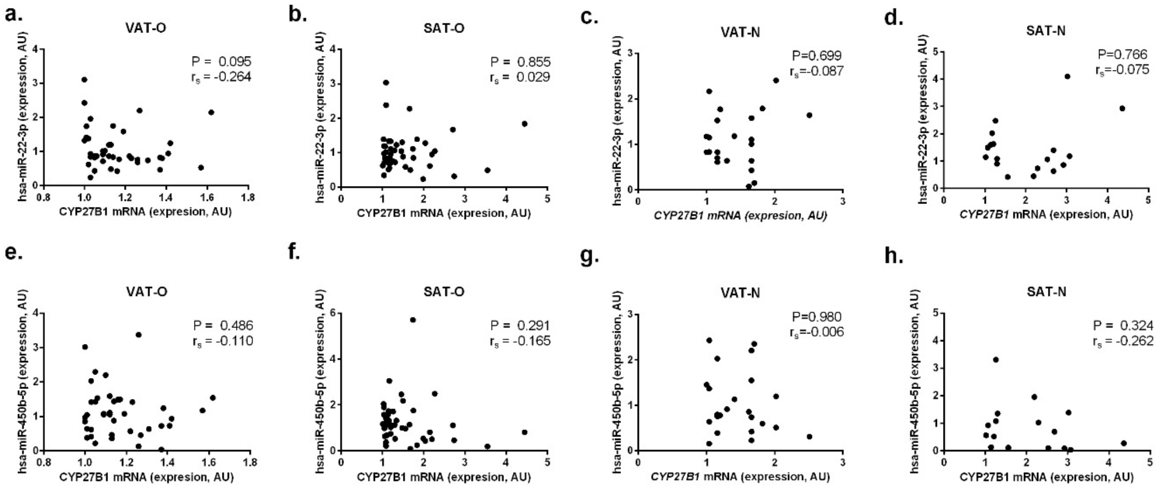

Supplementary Figure 3. Correlation between mRNA levels of *CYP27B1* and expression of hsa-miR-22-3p (a, b, c, d) and hsa-miR-450b-5p (e, f, g, h) in visceral (VAT) and subcutaneous (SAT) adipose tissues of obese (O) and normal-weight (N) individuals.

Supplementary Table 1. microRNAs potentially interacting with *VDR* and *CYP27B1* 3′UTR sequences selected based on the *in silico* analysis and results of the next-generation-sequencing.

VDR	***CYP27B1***
hsa-miR-125a-5p	hsa-miR-22-3p
hsa-miR-125b-5p	hsa-miR-335-3p
hsa-miR-214-3p	hsa-miR-450b-5p
hsa-miR-223-3p	hsa-miR-576-5p
hsa-miR-361-3p	hsa-miR-589-5p
hsa-miR-382-3p	hsa-miR-4662a-5p
hsa-miR-495-3p	

Supplementary Table 2. Real-time PCR conditions used for the expression analysis

Gene	Gene Description		Primers	Annealing (°C)
VDR	vitamin D receptor	F	5'-CATGAAGCGGAAGGCACTAT-3'	61
		R	5'-ATGTCCACACAGCGTTTGAG-3'	
CYP27B1	vitamin D 1α-hydroxylase	F	5'-TACCAGAGCCTCCCGGAAC-3'	64
		R	5'- AACAGCGTGGACACAAACAC-3'	
CYP24A1	vitamin D 24-hydroxylase	F	5'-CCTGCTGCCAGATTCTCTGGAA-3'	55
		R	5'-TTGCCATACTTCTTGTGGTACTCC-3'	
IL1B	interleukin 1β	F	5'-CACCAAGCTTTTTTGCTGTGAGT-3'	60
		R	5'-GCACGATGCACCTGTACGAT-3'	
IL6	interleukin 6	F	5'-CCTTCGGTCCAGTTGCCTTC-3'	60
		R	5'-GTGGGGCGGCTACATCTTTG-3'	
IL8	interleukin 8	F	5'-CACCGGAAGAACCATCTCACT-3'	60
		R	5'-TCAGCCCTCTTCAAAAACTTCTCC-3'	
ACTB	β-actin	F	5'-CAGCCTGGATAGCAACGTAC-3'	61
		R	5'-TTCTACAATGAGCTGCGTGTG-3'	

F: forward primer; R: reverse primer.
